# Peer Review of “COVID-19 Infection and Symptoms Among Emergency Medicine Residents and Fellows in an Urban Academic Hospital Setting: Cross-sectional Questionnaire Study”

**DOI:** 10.2196/36209

**Published:** 2022-01-27

**Authors:** Jorge Tavares

**Affiliations:** 1 NOVA Information Management School Universidade NOVA de Lisboa Lisboa Portugal

**Keywords:** COVID-19, emergency medicine, housestaff wellness, medical education, training, frontline health care workers, frontline, personal protective equipment, pandemic, infectious disease, emergency

Round 1 Review

General Comments

This is a well-written paper [1] focusing on a very relevant topic. More details should be provided about the statistical approach used.

Specific Comments

It is a fact that the sample size is small and that is probably the reason why the Fisher exact test was used. Nonetheless, no explanation or rationale for the decision is given.

Major Comments

1. The sample size of this study is small and that seems to be the reason for using the Fisher exact test; however, more rationale should be provided for this decision, to explain if the assumptions required to perform the chi-square test are complied to or not. Another option for small samples is to use the Monte Carlo Simulation; why have you decided not to use that instead of the Fisher exact test?

Minor Comments

2. For such a small sample, you have a lot of categories pertaining to the number of patients treated (Table 2). In fact, these categories contributed little to provide significant results; have you tried to run the analysis with fewer categories? Can you please explain the rationale for having so many categories for a small sample?

3. I understand that the sample is small, and you are limited by it and by the characteristics of the variables, but have you tried to explore other types of statistical analysis and variables, using, for example, age or gender, with polymerase chain reaction (PCR) test rates, etc?

4. In Table 2, it is mentioned that the values for *P*<.05 are in bold; one value is .05 and it is bolded—are you talking about rounding in terms of the number of decimals? Was this result statistically significant according to SPSS?

Abbreviations

PCR: polymerase chain reaction
